# 2241. Antibiotic Use Before and After Hospital Admission Among Hospitalized Patients with Acute Febrile Illness in Bangladesh

**DOI:** 10.1093/ofid/ofad500.1863

**Published:** 2023-11-27

**Authors:** Fahmida Chowdhury, Tanzir Ahmed Shuvo, Anik Palit, Mohammed Ziaur Rahman, Robir Kumar Ghosh, Pawan Angra, Matthew Ikoleit, Arpita Shyama Deb, Muntasir Alam, Daniel W Martin, Mahmudur Rahman

**Affiliations:** icddr,b, Dhaka, Dhaka, Bangladesh; icddr,b, Dhaka, Dhaka, Bangladesh; icddr,b, Dhaka, Dhaka, Bangladesh; icddr,b, Dhaka, Dhaka, Bangladesh; icddr,b, Dhaka, Dhaka, Bangladesh; US CDC, Atlanta, Georgia; US CDC, Atlanta, Georgia; icddr,b, Dhaka, Dhaka, Bangladesh; icddr,b, Dhaka, Dhaka, Bangladesh; Centers for Disease Control and Prevention, Atlanta, GA; GHD EMPHNET, Dhaka, Dhaka, Bangladesh

## Abstract

**Background:**

The inappropriate use of antibiotics is common in Bangladesh, contributing to the significant burden of antimicrobial resistance. The WHO has categorized Access, Watch, and Reserve groups of antibiotics (AWaRe classification) to promote optimal use. We explored antibiotic use among hospitalized patients with acute febrile illness (AFI) at five tertiary-level hospitals in Bangladesh.

**Methods:**

From September 2021 to February 2023, 1544 patients were randomly selected among all hospitalized patients with measured fever (≥100.4°F) or a history of fever in the past 14 days. Blood and urine culture isolates were analyzed for antibiotic sensitivity using the VITEK-2 system.

**Results:**

Of enrolled AFI patients, 835 (54%) reported the use of antibiotics prior to hospitalization, with 79% of reported antibiotics from the Watch group, 16% from the Access group, and none from the Reserve group. Five percent of the antibiotics prescribed were not recommended by WHO for clinical practice. The top five frequently reported antibiotics before hospitalization were cefixime (24%), azithromycin (19%), ceftriaxone (16%), amoxicillin (6%), and cefuroxime (5%). Bacterial infection was diagnosed among 205 patients (13% of all enrollees) through Rapid diagnostic tests, culture and Rt-PCR. In-hospital, 1385 (90%) patients received at least one antibiotic. The most frequently prescribed antibiotics were from the Watch group (84%), followed by the Access group (15%) and none from the Reserve group. The top five antibiotics prescribed in-hospital were ceftriaxone (64%), amoxiclav (7%), ceftazidime (3.4%), clarithromycin (2.8%), and moxifloxacin (2.6%). Antibiotic susceptibility testing (AST) results from blood and urine culture isolates showed high resistance pattern to ceftriaxone (45% and 56%) and ceftazidime (63% and 50%). Of blood culture isolates 80% were sensitive to amoxiclav, while 46% of urine culture isolates were resistant to amoxiclav. At least one bacterial organism was identified in only 13% of enrolled patients.

List of antibiotics that were reported to be received by the enrolled AFI patients before Hospitalization

**Figure:**
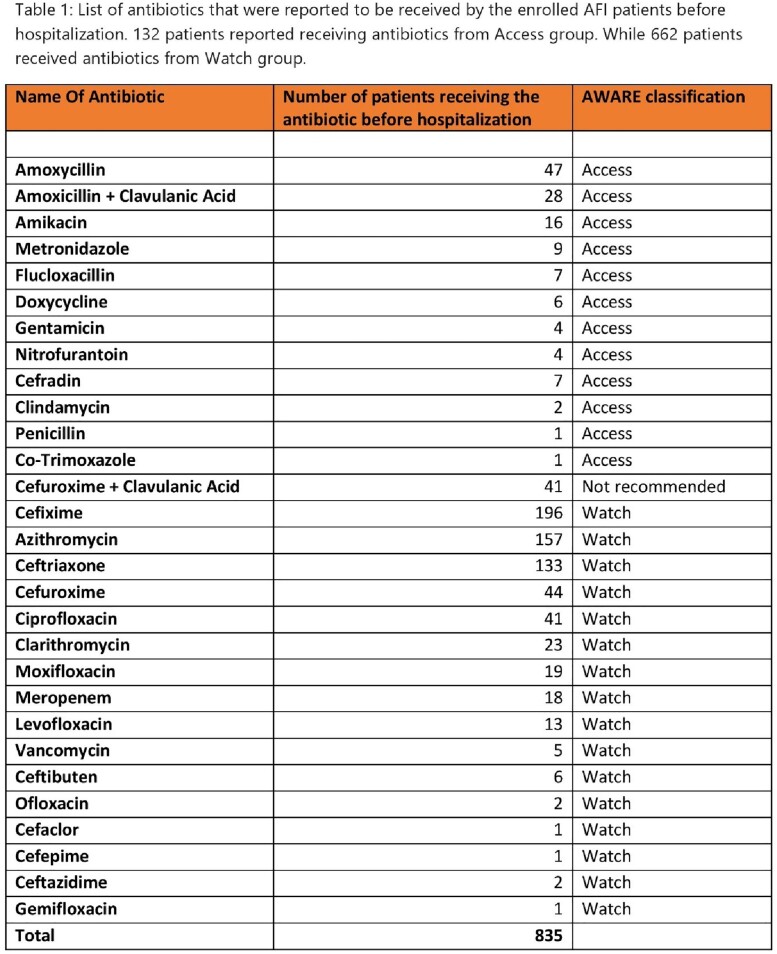

Before hospitalization, 132 patients reported receiving antibiotics from Access group, while 662 patients received antibiotics from WATCH group

List of antibiotics received by the enrolled AFI patients during hospitalization

**Figure:**
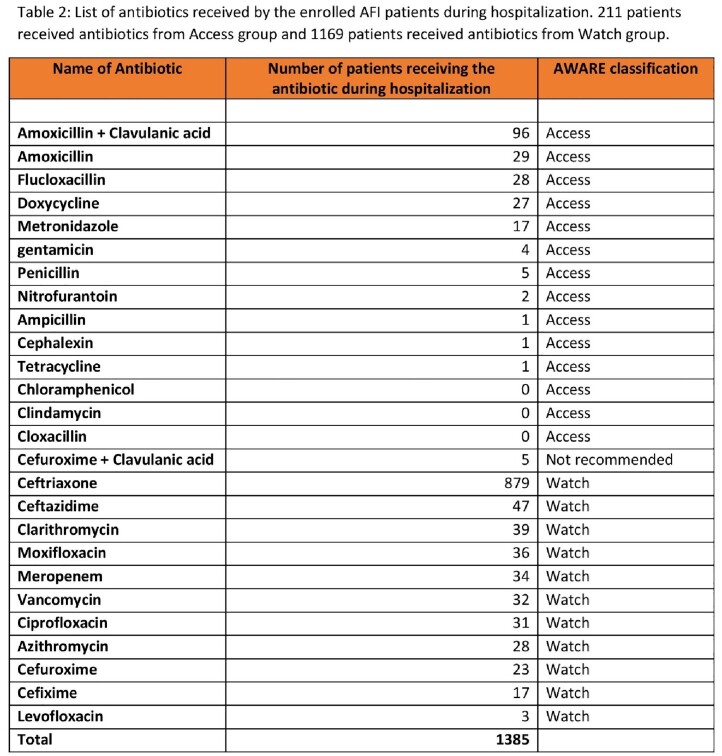

During hospitalization, 211 patients received antibiotics from Access group and 1169 patients received antibiotics from Watch Group

List of 235 bactrial organisms which were identified from clinical samples of 205 AFI patients (some patient had co-infection)

**Figure:**
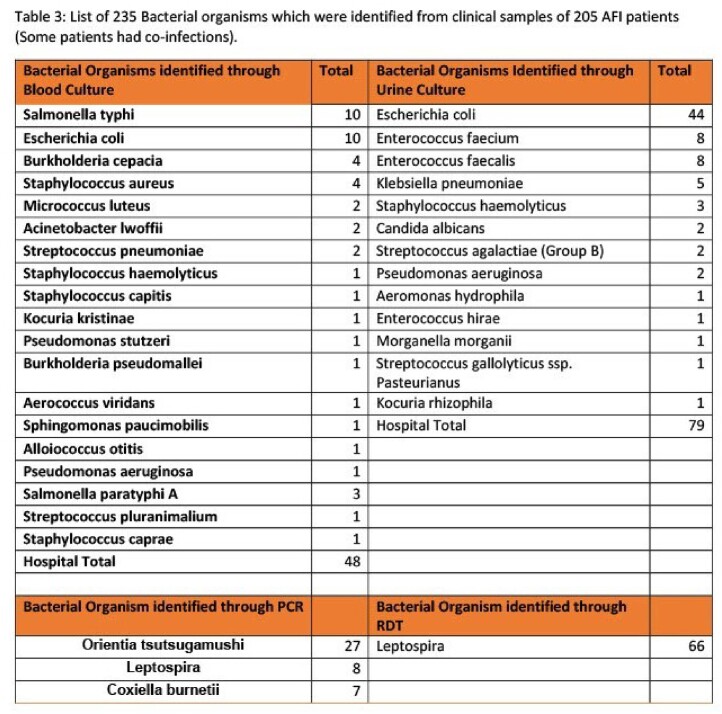

**Conclusion:**

In conclusion, antibiotics were used in nine of ten patients. The frequent and inappropriate use of Watch group antibiotics in outpatient settings likely contributes to decreased effectiveness and demonstrates the need for improved antimicrobial stewardship.

**Disclosures:**

**All Authors**: No reported disclosures

